# Artificial intelligence for surgical outcome prediction in glaucoma: a systematic review

**DOI:** 10.3389/fdata.2025.1605018

**Published:** 2025-08-08

**Authors:** Zeena Kailani, Lauren Kim, Joshua Bierbrier, Michael Balas, David J. Mathew

**Affiliations:** ^1^Michael G. DeGroote School of Medicine, McMaster University, Hamilton, ON, Canada; ^2^Queen's School of Medicine, Queen's University, Kingston, ON, Canada; ^3^Department of Ophthalmology and Vision Sciences, University of Toronto, Toronto, ON, Canada; ^4^Donald K. Johnson Eye Institute, Krembil Research Institute, University Health Network, Toronto, ON, Canada

**Keywords:** glaucoma, trabeculectomy, glaucoma surgery, artificial intelligence, machine learning, deep learning, surgical outcomes, outcome prediction

## Abstract

**Introduction:**

Glaucoma is a leading cause of irreversible blindness, and its rising global prevalence has led to a significant increase in glaucoma surgeries. However, predicting postoperative outcomes remains challenging due to the complex interplay of patient factors, surgical techniques, and postoperative care. Artificial intelligence (AI) has emerged as a promising tool for enhancing predictive accuracy in clinical decision-making.

**Methods:**

This systematic review was conducted to evaluate the current evidence on the use of AI to predict surgical outcomes in glaucoma patients. A comprehensive search of Medline, Embase, Web of Science, and Scopus was performed. Studies were included if they applied AI models to glaucoma surgery outcome prediction.

**Results:**

Six studies met inclusion criteria, collectively analyzing 4,630 surgeries. A variety of algorithms were applied, including random forests, support vector machines, and neural networks. Overall, AI models consistently outperformed traditional statistical approaches, with the best-performing model achieving an accuracy of 87.5%. Key predictors of outcomes included demographic factors (e.g., age), systemic health indicators (e.g., smoking status and body mass index), and ophthalmic parameters (e.g., baseline intraocular pressure, central corneal thickness, mitomycin C use).

**Discussion:**

While AI models demonstrated superior performance to traditional statistical approaches, the lack of external validation and standardized surgical success definitions limit their clinical applicability. This review highlights both the promise and the current limitations of artificial intelligence in glaucoma surgery outcome prediction, emphasizing the need for prospective, multicenter studies, publicly available datasets, and standardized evaluation metrics to enhance the generalizability and clinical utility of future models.

**Systematic review registration:**

https://www.crd.york.ac.uk/PROSPERO/view/CRD42024621758, identifier: CRD42024621758.

## Introduction

Glaucoma remains a leading cause of irreversible blindness worldwide, with 60.5 million individuals affected in 2010 and global prevalence expected to reach 111.8 million by 2040 (Flaxman et al., 2017). As the global burden of disease grows, the number of glaucoma surgeries continues to rise. In the United States alone, the annual volume of glaucoma surgeries significantly increased by 176.7%, rising from 80,151 procedures in 2011 to 221,602 procedures in 2021 (Jayaram et al., 2024).

Although surgery plays a critical role in glaucoma management, postoperative outcomes are notoriously variable, influenced by a complex interplay of patient factors, surgical technique, and postoperative care. Complications such as hypotony maculopathy and bleb-related endophthalmitis pose significant risks to vision and quality of life, while also placing substantial burdens on healthcare systems due to prolonged follow-up, additional interventions, and higher costs (Vijaya et al., 2011; Stokes et al., 2022). This variability has led researchers to develop predictive models aimed at identifying patients at higher risk of complications, traditionally using logistic regression and other conventional statistical techniques (Lavin et al., 1992; Jampel et al., 2001; Lehmann et al., 2000; Parrish et al., 2001).

However, these traditional models are inherently limited by their reliance on linear assumptions and a small set of predefined variables, meaning they often struggle to capture the complex, nonlinear relationships that may drive surgical success or failure. In contrast, artificial intelligence (AI) models offer far more flexible and adaptive approaches. AI models can capture complex, nonlinear relationships in surgical outcomes that traditional statistical methods often overlook (Hashimoto et al., 2018). In addition, they can continuously learn and evolve, incorporating new data over time to improve their predictive accuracy and remain clinically relevant (Huang et al., 2018).

AI has already demonstrated considerable success in other areas of ophthalmology, including automated detection of diabetic retinopathy, prediction of visual field progression, and detection of glaucoma itself using fundus photography and optical coherence tomography (OCT) (Lee and Lee, 2020; Giannini et al., 2019). Its application to surgical outcome prediction, however, represents a more recent development; one that holds great potential to support personalized surgical planning and targeted postoperative care.

The successful translation of these predictive models into routine surgical care depends not only on their accuracy but also on their clinical interpretability, generalizability across patient populations, and external validation. To better understand the current state of this evolving field, we conducted a systematic review to comprehensively evaluate the scientific evidence on AI-driven prediction of postoperative outcomes in glaucoma surgery. The review aims to assess the quality, performance, and limitations of current models, with the ultimate goal of identifying opportunities for improvement and priorities for future research.

## Methods

### Study design

This systematic review was conducted to evaluate the application of AI in predicting postoperative outcomes following glaucoma surgery. To ensure methodological transparency and comprehensive reporting, we adhered to the Preferred Reporting Items for Systematic Reviews and Meta-Analyses (PRISMA) guidelines (Moher et al., 2009).

### Search strategy

A comprehensive search of the literature was performed across four major databases: Medline, Embase, Web of Science, and Scopus. The search covered the period from inception to November 20, 2024. The search strategy incorporated terms related to AI, machine learning, deep learning, glaucoma surgery, trabeculectomy, and surgical outcomes. Publication date and language were not restricted. The complete search strings for each database are available in Supplementary Table 1.

### Eligibility criteria

Studies were eligible for inclusion if they met the following criteria: they were human studies with a cohort, cross-sectional, observational, or randomized controlled trial design; they examined the use of AI to predict or assess postoperative outcomes following glaucoma surgery; and they were published in English. Studies were excluded if they were systematic reviews, scoping reviews, case reports, letters to the editor, abstracts, or conference posters. Animal studies, studies without relevant outcomes or outcome data, and studies published in languages other than English were also excluded.

### Screening and study selection

Two independent reviewers (ZK and LK) screened the titles and abstracts of all citations for eligibility based on the predefined inclusion and exclusion criteria. Full-text review was performed for all studies that met initial screening criteria. Any disagreements during either stage of screening were resolved through consensus discussion between the two reviewers. If consensus could not be reached, a third reviewer served as an arbiter. After full-text review, six studies remained and were selected for final inclusion and data extraction. The study selection process is summarized via a PRISMA flow chart in Supplementary Figure 1.

### Data extraction

Data extraction was conducted using a standardized spreadsheet developed a priori in Google Sheets (available in Supplementary Table 2). Two independent reviewers (ZK and LK) extracted data from each of the six included studies following a full-text review. All extracted data were compared between reviewers, and discrepancies were resolved through discussion. If necessary, a third reviewer (MB) was consulted to resolve any conflicts.

The extracted data included study authorship, year of publication, study objective, study design, setting (single-center or multicenter), sample size, mean age, and sex distribution of the study population. Data extraction also captured each study's inclusion and exclusion criteria, data source, type of surgical intervention, and definitions of surgical success and failure. Additionally, data on the outcomes predicted by the AI models, the types of algorithms employed, the methods used to develop and validate the models, and the performance of each model were collected. The specific input parameters or features used for prediction were recorded along with any reported feature importance rankings. Key conclusions from each study, any author-reported limitations, and all reported evaluation metrics, including accuracy, area under the receiver operating characteristic curve (AUROC), area under the precision-recall curve (AUPRC), anomaly correlation coefficient (ACC), Matthews correlation coefficient (MCC), F1 score, precision, negative predictive value (NPV), sensitivity, and specificity, were also extracted.

To ensure consistent interpretation of AUROC scores across studies, we applied the classification system proposed by Hosmer and Lemeshow (2000). AUROC values below 0.5 were categorized as “bad,” values from 0.5 to 0.7 as “poor,” values from 0.7 to 0.8 as “acceptable,” values from 0.8 to 0.9 as “excellent,” and values exceeding 0.9 as “outstanding.”

### Quality assessment

The methodological quality and risk of bias for all included studies were evaluated using the Joanna Briggs Institute (JBI) Critical Appraisal Checklist for Cohort Studies (Moola et al., 2020). Each study was independently appraised by two reviewers (ZK and LK). Any discrepancies in the initial assessments were discussed until consensus was reached. If necessary, a third reviewer (MB) was consulted.

### Data synthesis

A meta-analysis was not performed due to substantial heterogeneity across the included studies, including differences in the types of algorithms and models used, variability in how data and outcomes were presented, and inconsistency in the evaluation metrics applied. Instead, we conducted a narrative synthesis, with a focus on summarizing each study's methodological approach, algorithms, input features, and reported predictive performance.

### Study outcomes

This systematic review primarily examined how effectively AI algorithms predict postoperative outcomes in glaucoma surgery. Secondary outcomes included characterization of the specific algorithms and models used, the types of input data and features incorporated into model development, approaches to model training and validation, and the degree to which performance varied across different study designs, definitions of surgical success, and model architectures.

## Results

### Study characteristics

Our search yielded 91 studies; 39 studies underwent title and abstract screening after duplicate removal; 8 articles were selected for full-text review; 2 articles were removed, leaving a total of 6 studies. Our included studies collectively analyzed 4,630 glaucoma surgeries involving 3,523 unique patients. The median sample size across studies was 218.5 (IQR: 153 to 1,540), ranging from 102 (Barry and Wang, 2024) to 2,398 surgeries (Agnifili et al., 2023), with a median follow-up duration of 1 year (IQR: 0), spanning from 3 months (Barry and Wang, 2024) to 5 years (Birla et al., 2024). In terms of study design, four studies were retrospective cohort studies and two employed prospective cohort designs. Five studies relied on single-center data, with only one drawing from a multicenter database (Agnifili et al., 2023).

In terms of patient demographics, female patients comprised 48.9% (*n* = 1,723) of the total cohort. The average age across 4 studies reporting means was 68.0 years (pooled SD: 15.9), while 2 studies reported median ages of 74 (Agnifili et al., 2023) and 26 years Birla et al., (2024), respectively. In terms of racial representation across studies, 53.6% of the total sample were White, 19.1% were Asian, 8.9% were non-White Hispanic, 8.6% were unknown or unreported by the authors, 4.2% were Black, and 5.6% were of another race. None of the included studies reported the socioeconomic characteristics of their respective patient populations.

Most studies (*n* = 4) included patients with various subtypes of glaucoma, with three including both primary and secondary glaucoma. Birla et al. (2024) was the only study to focus exclusively on just one subtype [juvenile open-angle glaucoma (JOAG)]. Barry and Wang (2024) and Lin et al. (2024) did not report the specific diagnoses of 100% and 28% of their respective samples. Overall, four of the studies included patients with primary open-angle glaucoma (POAG), three included patients with pseudoexfoliative glaucoma (PXG), two included patients with JOAG, two included patients with primary angle-closure glaucoma (PACG), one included patients with pigment dispersion glaucoma (PDG), and one included patients with uveitic glaucoma (UG) and neovascular glaucoma (NVG). Additionally, the types of procedures varied among studies. Most (*n* = 4) focused exclusively on trabeculectomy, one focused on Ahmed valve implantation, and another took a broader approach and encompassed trabeculectomy, ExPress shunts, tube shunts, minimally invasive glaucoma surgery (MIGS), and cyclophotocoagulation (CPC).

The heterogeneity of patient populations, diagnoses, and procedures contributed to differences in success criteria and model features across the studies. Details of the included studies can be found in Table 1.

**Table 1:** Study characteristics.

**Study—first author(s) (year)**	**Study design**	**Sample size (eyes)**	**Age (years)**	**Female (%)**	**Race (%)**	**Procedure(s)**	**Glaucoma type(s)**	**Follow-up duration**
(Agnifili et al. 2023)	Prospective cohort study	102	74.0 (62.8–80.0)	42 (41.2)	N/R	Trabeculectomy	POAG, PXG	1 year
(Banna et al. 2022)	Retrospective cohort study	230	68.7 (±12.4)	95 (41.3)	White (90.9) Black (9.1)	Trabeculectomy	POAG, PXG, PDG, JOAG, chronic PACG	1 year
(Barry and Wang 2024)	Retrospective cohort study	2,398	72.5 (±15.4)	745 (47.4)	Asian (32.8) White (31.8) Hispanic (18.1) Other (10.7) Black (5.5) Unknown (1.1)	Trabeculectomy, ExPress shunts, tube shunts, MIGS, cyclophotocoagulation	N/R	3 months
(Birla et al. 2024)	Prospective cohort study	207	26 (10–39)	52 (33)	N/R	Trabeculectomy	JOAG	5 years
(Lee et al. 2024)	Retrospective cohort study	153	62.4 (±13.9)	46 (30.1)	Asian (100.0)	Ahmed valve implantation	POAG, UG, NVG, PXG, PACG	1 year
(Lin et al. 2024)	Retrospective cohort study	1,540	63.6 (±15.7)	879 (57.0)	White (86.0) Asian (4.0) Hispanic (4.0) Black (3.0) Other (3.0)	Trabeculectomy	POAG (72%)	1 year

POAG, primary open-angle glaucoma; PXG, pseudoexfoliative glaucoma; PDG, pigment dispersion glaucoma; JOAG, juvenile open-angle glaucoma; PACG, primary angle-closure glaucoma; MIGS, minimally invasive glaucoma surgery; UG, uveitic glaucoma; NVG, neovascular glaucoma.

### Algorithms and models

Across the six included studies, over 20 distinct algorithms were tested. The most frequently used algorithms included: random forests (*n* = 3), support vector machine (SVM, *n* = 3), decision trees (*n* = 2), and neural networks (*n* = 2). Other tested models included extreme gradient boosting (XGBoost), k-nearest neighbors, Bio-Clinical BERT, and Text Transformers, among others (Table 2). This wide range of models highlights both the exploratory nature of the field and the absence of a universally agreed-upon approach for surgical outcome prediction in glaucoma. Overall, the best performing models across studies were random forests (*n* = 2), decision trees (*n* = 2), XGBoost (*n* = 1), and a transformer multilayer neural network architecture (*n* = 1) (Table 3).

**Table 2:** Model characteristics from each included study.

**Study—first author(s) (year)**	**Models assessed**	**Best performing model**	**Features used**	**Validation method**	**Variable importance ranking**
(Agnifili et al. 2023)	Decision trees	N/A	Demographic characteristics, glaucoma-related clinical data, ocular surface features	N/R	CST; SCR; age; CET; ECR; ocular surface clinical test; surgical site-derived parameters
(Banna et al. 2022)	Random forest, SVM, MNN, multivariable logistic regression	Random forest (trained using demographic, ophthalmic, and systemic data)	Demographic characteristics, systemic health data, ocular parameters	Five-fold cross-validation	age; preoperative VA; ARB; MI; CCT; BMI; topical alpha-agonist; white race; smoking history; COPD; inhaled corticosteroids; preoperative IOP class (≥18 and ≤ 25); PGA; gender; number of preoperative medication classes (4 to 5); oral CAI; preoperative IOP class (>25); HTN; CVA; HLD
(Barry and Wang 2024)	Decision trees, random forest, XGBoost, penalized logistic regression, multi-layer perceptron, k-nearest neighbors, GNB, linear discriminant analysis, SVM	Random forest	Demographic characteristics, past ocular surgeries, diagnoses, medications, social history, ophthalmologic exam findings, concurrent cataract surgery on same day as glaucoma surgery, contact/glasses use	Five-fold cross-validation	IOP; trabeculectomy; concurrent cataract extraction; MIGS; tube shunt; RSE; acetazolamide; brimonidine tartrate; astigmatism; CCT; age; brimonidine tartrate/timolol; ARNC; presence of IOL; dorzolamide HCl; BCVA; glaucoma (unspecified); erythromycin; netarsudil mesylate
(Birla et al. 2024)	Functional trees, bagging, logistic regression	Functional trees	Demographic characteristics, ocular parameters	Ten-fold cross-validation	intraoperative MMC; age at diagnosis; tenon's thickness; baseline IOP; treatment duration; fistulization technique
(Lee et al. 2024)	Logistic regression, XGBoost, SVM	XGBoost (trained using demographic, ophthalmic, and systemic data)	Sociodemographic characteristics, ophthalmologic features, comorbid conditions, systemic medications, psychiatric medications	Three-fold cross-validation	age; valve in sulcus; used patch; statin; POAG diagnosis; preoperative topical medication
(Lin et al. 2024)	Random forest, transformer, MNN-LSTM, MNN-BioClinicalBERT, MNN-Transformer	MNN-Transformer	Demographic characteristics, glaucoma diagnosis, active preoperative medication use, chronic systemic diseases, ocular parameters	Five-fold cross-validation	N/R

N/A, not applicable; N/R, not reported; CST, conjunctival stromal thickness; SCR, stromal conjunctival reflectivity; CET, conjunctival epithelial thickness; ECR, epithelial conjunctival reflectivity; SVM, support vector machine; MNN, multilayer neural network; VA, visual acuity; ARB, angiotensin receptor blocker; MI, myocardial infarction; CCT, central corneal thickness; BMI, body mass index; COPD, chronic obstructive pulmonary disease; IOP, intraocular pressure; prostaglandin analog; CAI, carbonic anhydrase inhibitor; HTN, hypertension; CVA, cerebrovascular accident; HLD, hyperlipidemia; XGBoost, eXtreme Gradient Boosting; GNB, Gaussian Naive Bayes; MIGS, minimally invasive glaucoma surgery; RSE, refraction spherical equivalent; ARMC, age-related nuclear cataract; IOL, intraocular lens; BCVA, best corrected visual acuity; MMC, mitomycin C; XGBoost, Extreme Gradient Boosting; POAG, primary open-angle glaucoma; MNN-LSTM, long short-term memory multimodal model; MNN-BioClinicalBERT, BioClinical BERT multimodal model; MNN-Transformer, transformer multimodal model.

**Table 3:** Performance metrics of best performing models.

**Study—first author(s) (year)**	**Best model**	**Outcome(s)**	**Accuracy**	**AUROC**	**Sensitivity**	**Specificity**
(Agnifili et al. 2023)	Decision trees	Surgical failure vs. surgical success	N/R	0.784	N/R	N/R
(Banna et al. 2022)	Random forest (trained using demographic, ophthalmic, and systemic data)	Surgical failure vs. surgical success	0.65	0.68	0.44	0.86
(Barry and Wang 2024)	Random forest	Surgical failure based on IOP reduction, medication use, or need for additional surgery	0.755	0.767	0.955	0.223
(Birla et al. 2024)	Functional trees	Surgical success based on postoperative IOP or reduction in IOP	0.8747	0.926	0.874	0.804
(Lee et al. 2024)	XGBoost (trained using demographic, ophthalmic, and systemic data)	Surgical failure	0.844	0.782	0.714	0.868
(Lin et al. 2024)	MNN-Transformer	Surgical success, surgical failure due to elevated IOP, and surgical failure due to low IOP	0.735	0.750	0.659	0.811

AUROC, area under the receiver operating characteristic; XGBoost, extreme gradient boosting; MNN-Transformer, transformer multimodal model; IOP, intraocular pressure.

### Methodological quality and validation

All included studies were appraised using the JBI Critical Appraisal Checklist for Cohort Studies and were determined to be of high methodological quality (Table 4). Five of the six studies performed internal validation, with most using k-fold cross-validation (k = 3 to 10). External validation, a more rigorous test of generalizability, was reported in only one study (Birla et al., 2024), which tested its model on a separate set of five unrelated patients. However, a validation cohort of this size is insufficient to assess real-world performance across diverse patient populations and overestimates the model's predictive reliability.

**Table 4:** Quality appraisal using Joanna Briggs Research Institute Checklist for Cohort Studies.

**Study—first author(s) (year)**		**1. Were the two groups similar and recruited from the same population?**	**2. Were the exposures measured similarly to assign people to both exposed and unexposed groups?**	**3. Was the exposure measured in a valid and reliable way?**	**4. Were confounding factors identified?**	**5. Were strategies to deal with confounding factors stated?**	**6. Were the groups or participants free of the outcome at the start of the study (or at the moment of exposure)?**	**7. Were the outcomes measured in a valid and reliable way?**	**8. Was the follow up time reported and sufficient to be long enough for outcomes to occur?**	**9. Was follow up complete, and if not, were the reasons to loss to follow up described and explored?**	**10. Were strategies to address incomplete follow up utilized?**	**11. Was appropriate statistical analysis used?**	**Overall quality**
(Agnifili et al. 2023)													
(Banna et al. 2022)													
(Barry and Wang 2024)													
(Birla et al. 2024)													
(Lee et al. 2024)													
(Lin et al. 2024)													
Judgement:													
	Yes												
	No												
	N/A												

Color values in this table indicate the judgment of quality appraisal based on the Joanna Briggs Institute (JBI) Checklist. Green represents “Yes” (criterion met), red represents “No” (criterion not met), and gray represents “N/A” (not applicable).

### Management of class imbalance

Class imbalance, where one outcome (typically surgical failure) is far less common than the other, was directly addressed in three studies: Banna et al. (2022) applied up-sampling to equalize success and failure cases; Barry and Wang (2024) adjusted for imbalance through weighted loss functions, classification threshold optimization, and by tuning for both accuracy and F1 score; and Lin et al. (2024) fine-tuned models using macro-averaged AUROC and F1 score, both of which account for imbalanced data. The lack of standardization in addressing class imbalance (and not addressing it) contributed to variability in reported performance across studies and limits comparability.

### Surgical success criteria and outcome definitions

Surgical success was defined differently across the six studies, though most definitions centered on postoperative intraocular pressure (IOP) reduction. Some studies defined success as achieving IOP below an absolute threshold (e.g., <18 mmHg), while others required a percentage reduction from baseline. Beyond IOP, some studies expanded their definition of success to include factors such as avoidance of additional glaucoma surgery or reduced need for medication. Two studies also incorporated visual outcomes, specifically loss of light perception. Of note, the timing of success evaluation ranged from 3 months to 5 years postoperatively, with most studies requiring at least two consecutive follow-up visits before outcomes were classified. These inconsistencies in outcome definitions complicate direct performance comparisons and may have influenced the variability in reported AI model accuracy across studies.

### Model performance metrics

All six studies reported AUROC scores to evaluate their models' ability to distinguish between surgical success and failure. Five studies also reported accuracy, sensitivity, and specificity, and four reported precision scores. Additional metrics, including AUPRC (*n* = 3), F1 score (*n* = 3), MCC (*n* = 1), and NPV (*n* = 1), were reported in some cases.

The highest-performing model was developed by Birla et al. (2024), achieving an accuracy of 87.5% and an AUROC of 0.926. This model used a tree-based algorithm trained on both preoperative and intraoperative variables, including age at diagnosis, baseline intraocular pressure (IOP), duration of preoperative medical treatment, Tenon's thickness, intraoperative mitomycin C (MMC) administration, and scleral fistulation technique. Despite being trained on a relatively small dataset (*n* = 218), its performance suggests that effective feature selection may be more critical than dataset size alone.

In contrast, Lin et al. (2024) developed the least accurate model, a long short-term memory (LSTM)-based deep learning model trained exclusively on free-text operative notes, which achieved an accuracy of only 40.9%. This low performance is partially explained by the multi-class nature of the prediction task, which categorized outcomes into surgical success, failure due to elevated IOP, or failure due to low IOP. The poor accuracy highlights the challenges of using unstructured text data alone for predictive modeling in glaucoma surgery. However, when the LSTM model incorporated structured electronic health record (EHR) data, accuracy improved to 63.5%, reinforcing the importance of structured data for AI-based surgical outcome prediction.

Sensitivity and specificity varied widely across studies, with three studies reporting higher specificity than sensitivity, while two reported the opposite. Sensitivity ranged from 0.152 (Gaussian Naïve Bayes in Barry and Wang, 2024) to 1.000 (multiple models in Lee et al., 2024), while specificity ranged from 0.089 (decision tree in Barry and Wang, 2024) to 0.926 (Gaussian Naïve Bayes in Barry and Wang, 2024). Such extreme variability in performance metrics likely reflects differences in study populations, feature selection, dataset size, and model architecture. Models trained on single-center datasets generally reported higher accuracy, but this likely reflects overfitting to site-specific data rather than true predictive superiority. The lack of robust external validation in most studies raises concerns about their generalizability in real-world clinical practice.

## Discussion

### Overview of findings

To our knowledge, this is the first systematic review to evaluate the role of AI in predicting postoperative outcomes in glaucoma surgery. Our findings indicate that AI models hold promise for forecasting surgical outcomes, though performance varied widely across studies. The best-performing models achieved accuracy rates as high as 87.5% (Birla et al., 2024), while the weakest model yielded an accuracy of just 40.9% when predicting multi-class outcomes (Lin et al., 2024). Notably, tree-based algorithms such as random forests consistently ranked among the top-performing models, aligning with broader findings in surgical AI research where tree-based methods have demonstrated robust performance (Hassan et al., 2022).

Despite differences in modeling approaches, certain features emerged as consistently important for predictive accuracy. These included demographic factors (e.g., age), systemic health indicators [e.g., body mass index (BMI), smoking history, comorbid conditions], and ophthalmic parameters [e.g., central corneal thickness (CCT), preoperative intraocular pressure (IOP), preoperative visual acuity (VA), and active ocular medications]. The predominance of structured clinical data suggests that natural language processing (NLP) remains an underutilized tool in glaucoma surgery prediction, with only one study (Lin et al., 2024) incorporating unstructured operative notes. However, significant challenges remain, including the lack of standardized definitions for surgical success and limited external validation, both of which hinder the generalizability and clinical applicability of these models.

This review highlights both the promise and current limitations of AI in glaucoma surgery outcome prediction. While AI models offer a powerful tool for personalized surgical planning and risk stratification, their translation into clinical practice will require addressing key methodological and practical barriers, as explored in the following sections.

### Algorithm performance and key determinants of success

Comparing model performance across the included studies was challenging, largely due to variations in dataset size, validation protocols, and performance metrics. Nevertheless, several general observations emerged. First, model performance did not necessarily improve with larger sample sizes. For instance, Birla et al. (2024) achieved high accuracy despite training on just over 200 samples, which may reflect effective feature selection, a well-curated dataset, or possible overfitting. The limited sample size underscores the need for caution when drawing conclusions about model performance. In addition, models that included a broader range of features, particularly systemic health data, often exhibited superior performance (Banna et al., 2022; Lee et al., 2024; Lin et al., 2024).

A more meaningful comparison arises from studies that directly evaluated multiple models under uniform validation conditions. Random forest and XGBoost typically outperformed other algorithms (Banna et al., 2022; Barry and Wang, 2024; Lee et al., 2024; Lin et al., 2024). Barry and Wang (2024) reported that random forest models surpassed both XGBoost and logistic regression across nearly all validation metrics, highlighting the value of capturing nonlinear relationships in clinical data. Although sensitivity, specificity, and precision varied significantly across models, no single metric consistently distinguished a best model, suggesting that clinical priorities and data availability should inform model selection.

Random forests excelled particularly well in small-to-medium-sized datasets and multiclass prediction tasks, often outperforming more complex architectures when training data were limited (Banna et al., 2022; Barry and Wang, 2024; Birla et al., 2024). These ensemble-based models are also known to mitigate overfitting (Banna et al., 2022; Merali et al., 2019) and have shown successful applications in electronic medical record analyses (Merali et al., 2019; Tseng et al., 2020). Indeed, tree-based models have frequently outperformed deep learning methods in tabular medical datasets, particularly when the sample size is modest (Grinsztajn et al., 2022; Shwartz-Ziv and Armon, 2022). However, these findings may not extrapolate to scenarios involving substantially larger datasets or high-dimensional feature modalities such as imaging or textual data, where deep learning often has an advantage (Shwartz-Ziv and Armon, 2022).

Deep learning models, including neural networks, Transformers, and LSTMs, tended to underperform in these studies, likely due to insufficient training samples. For example, Barry and Wang (2024) reported that a neural network trained on over 2,000 cases performed comparably to their top tree-based model, whereas Banna et al. (2022) found that a neural network trained on fewer samples fared worse. In addition, tree-based approaches are inherently less suited to unstructured data (e.g., images or text), where deep learning methods are typically favored. Lin et al. (2024) explored Transformers and LSTMs for analyzing surgeon operative notes, but achieved suboptimal accuracy (<50%). This may reflect either limited training data or an insufficiently discriminative textual feature set. Nonetheless, performance improved when structured EHR data were combined with unstructured text, implying that multimodal approaches may be more effective than relying exclusively on text-based inputs.

Lastly, no studies investigated regression-based approaches to predict continuous variables such as postoperative IOP, a strategy that could potentially yield more nuanced clinical insights. Whether a study adopts a classification or regression framework ultimately depends on clinical priorities and the nature of the available data. While classification models are useful for binary or multiclass outcomes (e.g., success vs. failure), regression models might better capture gradations in postoperative results, thus supporting more personalized surgical planning.

### The importance of feature selection

Despite inconsistent reporting of feature importance rankings across studies, we were still able to conclude that feature selection plays a critical role in model performance, a finding that aligns with prior research on AI algorithms (Pudjihartono et al., 2022; Wang et al., 2024). Across our included studies, a range of preoperative, intraoperative, and postoperative variables were found to be of high predictive value. Among preoperative variables, age, baseline IOP, CCT, and active topical medications emerged as key discriminative factors. Age was the most consistently highly ranked variable, though its association with surgical success or failure varied across studies, underscoring its biological relevance even if its predictive direction remains inconsistent. Baseline IOP, a well-established clinical predictor, was heavily relied upon by AI models, with higher preoperative IOP often associated with greater postoperative reduction. This reinforces its importance in preoperative risk stratification and surgical planning. Less traditional factors, such as smoking history and body mass index (BMI), also demonstrated strong discriminative power, suggesting that a comprehensive preoperative assessment, incorporating both established and AI-identified risk factors, could enhance patient selection and surgical planning.

While most models relied on preoperative clinical data, such as age, baseline IOP, CCT, and past ocular surgeries, the inclusion of intraoperative and early postoperative factors further enhanced predictive accuracy. For example, MMC administration, surgical technique, and concurrent cataract extraction were notable predictors in studies that included intraoperative variables. These findings highlight the importance of capturing real-time surgical and recovery data to improve predictive power. Postoperatively, early IOP reduction and visual acuity trends were consistently identified as critical predictors of long-term outcomes. The reliance on early postoperative IOP aligns with clinical evidence that early IOP trends strongly predict surgical success or failure (Esfandiari et al., 2017; Rong et al., 2013). This finding reinforces the importance of close postoperative monitoring and timely interventions, such as early suture lysis or medication adjustments, to mitigate risks of failure. By identifying patients at higher risk based on early IOP trajectories, AI-assisted decision support could enable more proactive and personalized management strategies, ultimately improving long-term outcomes.

Although incorporating intraoperative and early postoperative variables enhances model performance, their inclusion may not always be clinically practical. In real-world applications, if the primary objective of these models is to assess a patient's suitability for surgery, these features would not be available at the time of preoperative evaluation. However, predictive models may also serve a role in immediate postoperative management, where these features could provide valuable insights. Thus, while their inclusion may not be essential for preoperative decision-making, it remains clinically relevant in models designed to support early postoperative monitoring and intervention. Overall, feature selection plays a critical role in model performance, with both traditional and AI-identified risk factors contributing to predictive accuracy.

### Implications for surgical practice

AI-driven predictions in glaucoma surgery have the potential to transform clinical practice by enabling personalized surgical planning, targeted postoperative monitoring, and proactive management strategies. By identifying patients at higher risk of surgical failure, such as those with thin corneas, low baseline IOP, or poor conjunctival health, AI models can guide surgeons toward alternative procedures such as tube shunts or adjunctive therapies such as MMC to optimize outcomes. Additionally, insights into intraoperative factors like scleral flap design and Tenon's thickness can help refine surgical techniques, improving wound healing and bleb formation.

Postoperatively, AI models can enhance patient care by flagging high-risk individuals for closer monitoring. For example, patients with poor early IOP control or declining visual acuity may benefit from timely interventions such as early suture lysis, bleb needling, or medication adjustments. This proactive approach can reduce the need for reoperations and improve long-term outcomes. Furthermore, AI-driven risk stratification can support patient counseling, helping clinicians set realistic expectations and tailor treatment plans based on individual risk profiles.

To maximize clinical utility, AI models may be integrated into EHRs to provide real-time decision support during preoperative planning and postoperative care. Specific use scenarios include preoperative decision support to assess surgical candidacy and guide procedure selection, identification of high-risk patients who might benefit from increased monitoring or adjunctive treatments, and adaptive follow-up planning to allocate resources more effectively based on individualized risk stratification. Automated alerts for high-risk patients can prompt clinicians to take proactive measures, while benchmarking surgical outcomes across institutions can identify best practices and areas for improvement. By leveraging AI-driven predictions, clinicians can enhance surgical outcomes, improve patient satisfaction, and reduce the burden of glaucoma-related vision loss.

### Limitations

Despite offering valuable insights into the emerging role of AI for predicting surgical outcomes in glaucoma, this review has several notable limitations. First, the small number of included studies, combined with their heterogeneous designs, datasets, and outcome definition, limits the ability to draw definitive conclusions or compare model performance across different clinical settings. Most of the studies were single-center and retrospective, raising concerns about potential overfitting to local patient populations; moreover, nearly all lacked robust external validation, making it difficult to assess generalizability to broader populations. The limited sample sizes in certain studies further amplify the risk of overfitting, especially when complex models, such as deep learning architectures, are applied. In addition, many of the datasets in included studies were imbalanced with respect to key demographic variables such as ethnicity and age, which not only undermines model performance in underrepresented groups but also raises broader concerns in respect to potential biases and inequities. The use of imbalanced data in automated decision-making systems may inadvertently perpetuate or exacerbate existing disparities in healthcare, and as AI models begin to influence clinical decision-making, these ethical implications must be addressed. This underscores the need for transparent model development, demographic diversity in training datasets, and ongoing bias monitoring in real-world implementation.

Second, definitions of “surgical success” and the selection of predictive features varied markedly among the included studies, contributing to inconsistencies in reported performance metrics. Although this variety reflects real-world differences in surgical practice and patient populations, it also underscores the need for standardized outcome definitions and comprehensive multicenter datasets. Additionally, while class imbalance was addressed in some studies, no uniform approach was used, which may obscure the true performance of AI models in predicting rare outcomes. Finally, the exclusion of non-English articles and conference abstracts may have led to publication bias, as relevant unpublished or non–peer-reviewed data were not incorporated. Together, these limitations underscore the need for larger, prospective, and externally validated studies with uniform definitions and standardized data collection to better establish the clinical utility of AI in glaucoma surgery.

### Future directions and testable theories

A primary goal for advancing AI in glaucoma surgery is to build comprehensive, high-quality datasets that capture preoperative, intraoperative, and postoperative variables. These data should include clinically relevant predictors, such as baseline IOP, CCT, and early postoperative IOP trends, while also integrating operative notes and imaging modalities like OCT. For example, Birla et al. (2024) demonstrated that a model incorporating intraoperative factors such as mitomycin C administration, Tenon's thickness, and scleral fistulation technique achieved an AUROC of 0.926, suggesting that inclusion of surgical detail can meaningfully enhance predictive accuracy. Similarly, Banna et al. (2022) and Lee et al. (2024) identified systemic health factors such as BMI, smoking history, and medication use as influential, underscoring the need to capture a broad set of patient-level features. Standardizing success and failure definitions across studies will further enable meaningful comparisons and consistent benchmarking of new models. In the included studies, definitions varied significantly, ranging from absolute IOP thresholds to percent reductions and avoidance of additional interventions, introducing variability in performance metrics. Adopting consistent use of the term “success” is critical due to its influence on model training. Thus, prospective clinical trials should adopt uniform, clinically relevant composite endpoints, such as those recommended by the American Academy of Ophthalmology's Glaucoma Preferred Practice Pattern^®^ Committee (Gedde et al., 2025) to support model generalizability. Furthermore, approaches to missing data and class imbalance, such as those methods proven effective in our review [e.g., up-sampling (Banna et al., 2022), weighted loss functions (Barry and Wang, 2024), and macro-averaged metrics (Lin et al., 2024)], should be explicitly reported to mitigate potential biases. In addition, large, multicenter datasets should be made publicly available to foster transparency and external validation while maintaining diligent de-identification protocols for patients' health information. The improved performance seen in single-center datasets in this review may reflect overfitting rather than generalizability, highlighting the importance of robust external testing in future work.

In parallel, there is a need to systematically compare deep learning models to more traditional machine learning methods (e.g., random forests, XGBoost) on large, multimodal datasets that include unstructured text and imaging. While random forests consistently outperformed deep models in most studies reviewed, such as those by Barry and Wang (2024) and Banna et al. (2022), accuracy improved when structured and unstructured data were combined, as demonstrated by Lin et al. (2024), where the model's performance increased from 40.9% to 63.5%. These findings should guide future trials exploring multimodal model development. Furthermore, rigorous validation, particularly external validation on independent, multicenter cohorts, is essential to establish real-world generalizability. Clear reporting of metrics such as AUROC, precision, and F1 score can clarify trade-offs between false positives and negatives, especially in imbalanced datasets. Moving forward, prospective clinical trials should evaluate whether AI-guided risk predictions improve surgeon decision-making and patient outcomes beyond standard care. For instance, models identifying patients at risk based on early postoperative IOP trends, demonstrated in multiple studies as a key predictor, could be tested for their ability to prompt earlier interventions such as suture lysis or medication adjustments. Moreover, real-time AI integration into clinical workflows (e.g., EHR-based alerts) represents a valuable testable theory: does instantaneous feedback to surgeons enhance postoperative results? In the future, this may be assessed through EHR-integrated systems that automatically redact personal health information from model inputs, audit all data accesses, and require clinician confirmation before displaying predictions to ensure patient privacy is maintained. We suggest that future studies attempt to evaluate the impact of AI predictive models on real outcomes in glaucoma patients, such as through RCTs where AI guides treatment decisions.

Another critical challenge is model interpretability. Strategies like SHapley Additive exPlanations (SHAP) can offer transparent insights into feature importance and help clinicians understand model outputs, potentially improving user trust and accelerating adoption. As the field evolves, continuous learning mechanisms may be incorporated to keep models updated with emerging surgical techniques and shifting patient demographics. Researchers could also explore regression-based approaches for predicting continuous outcomes such as exact postoperative IOP, which might provide richer, more personalized insights than binary classifications. Examining the clinical impact of these more granular predictions, especially in dynamic healthcare environments, remains an important direction for future investigations.

In addition to methodological advancements, several practical barriers must be addressed before AI can be deployed for clinical use. These include the need for intuitive, user-friendly interfaces that seamlessly integrate into clinical workflows, interoperability with EHRs, and adequate training for clinical staff to interpret and use AI-generated insights. Without addressing these operational factors, even the most highly accurate models may struggle to gain traction in real-world practice among ophthalmologists. In addition, patient privacy is essential, and federated learning approaches, which enable collaborative model training across institutions without requiring patient-level data sharing, may serve to enhance data privacy and regulatory compliance. “Finally, before AI-based predictive models can be approved for use as medical devices, they must meet regulatory requirements set by authorities such as the United States Food and Drug Administration (FDA) and obtain Conformité Européenne (CE) marking in Europe. For FDA approval, criteria include prospective clinical validation, transparency and interpretability of the model's decision-making process, external validation and demonstration of generalizability, robust risk analysis, a clearly defined intended use (e.g., preoperative decision support, identification of high-risk patients for intensified care, or adaptive follow-up planning), seamless integration into clinical workflows, and adherence to privacy and cybersecurity standards (U.S. Food Drug Administration, 2021, 2022). Similarly, CE marking in Europe requires formal clinical evaluation demonstrating real-world clinical benefit, submission of comprehensive technical documentation, appropriate risk classification, and evidence of generalizability and bias mitigation across diverse patient populations (European Parliament Council of the European Union, 2017).

To accelerate external validation while promoting equitable, generalizable AI development, we encourage glaucoma centers, research institutions, and developers to partner in creating multi-institutional data-sharing consortia. Such collaboration is critical as we look toward building diverse, high-quality datasets and in turn, advancing the responsible translation of predictive models into clinical care.

## Conclusion

The application of AI to predicting postoperative outcomes in glaucoma surgery is a promising but immature field. While the reviewed studies demonstrate clear potential, this potential remains limited by small datasets, inconsistent feature selection, and a lack of external validation. To fully realize the benefits of AI, future research must focus on standardization, external validation, prospective evaluation, and clinical impact assessment. Prospective trials evaluating AI integration into real-world surgical decision-making and postoperative care are also essential. Thus, although it is evident that AI already demonstrates potential in this field, without shared standards and robust validation, it remains at the “proof of concept” level. Addressing these methodological gaps will enable AI to fulfill its promise of enhancing personalized glaucoma surgery and improving patient outcomes.

## Data Availability

The original contributions presented in the study are included in the article/Supplementary material, further inquiries can be directed to the corresponding author.
