# Supplementary material for: Artificial intelligence for surgical outcome prediction in glaucoma: a systematic review
Source: Front Big Data. 2025 Aug 8;8:1605018. doi: 10.3389/fdata.2025.1605018 (PMC12370750; doi:10.3389/fdata.2025.1605018)
Supplement: Supplementary file 1 [file Table_1.docx]

**Supplementary Table 1.** Search Strategy

| # | Query | Results from Nov 20, 2024 |
| --- | --- | --- |
| **OVID Medline Epub Ahead of Print, In-Process & Other Non-Indexed Citations, Ovid MEDLINE(R) Daily and Ovid MEDLINE(R) 1946 to Present** <1946 to DATE> | | |
| 1 | exp Glaucoma/ | 62,134 |
| 2 | glaucoma*.tw,kf. | 76,287 |
| 3 | 1 or 2 | 86,942 |
| 4 | (trabeculectomy* or (glaucoma adj2 surg*) or migs).tw,kf. | 11,493 |
| 5 | 3 and 4 | 10,683 |
| 6 | outcome*.tw,kf. | 2,648,297 |
| 7 | 5 and 6 | 3,329 |
| 8 | exp Artificial Intelligence/ | 215,194 |
| 9 | exp Machine Learning/ | 79,756 |
| 10 | exp Deep Learning/ | 24,203 |
| 11 | ((artificial adj1 intelligence*) or ((machine or deep) adj1 learning) or AI).tw,kf. | 261,532 |
| 12 | 8 or 9 or 10 or 11 | 375,148 |
| 13 | 7 and 12 | 12 |
| **Embase** <1974 to DATE> | | |
| # | Query | Results from Nov 20, 2024 |
| 1 | exp Glaucoma/ | 107699 |
| 2 | glaucoma*.tw,kf. | 92565 |
| 3 | 1 or 2 | 122018 |
| 4 | (trabeculectomy* or (glaucoma adj2 surg*) or migs).tw,kf. | 14387 |
| 5 | 3 and 4 | 13116 |
| 6 | outcome*.tw,kf. | 3916728 |
| 7 | 5 and 6 | 4152 |
| 8 | exp Artificial Intelligence/ | 121060 |
| 9 | exp Machine Learning/ | 530969 |
| 10 | exp Deep Learning/ | 67985 |
| 11 | ((artificial adj1 intelligence*) or ((machine or deep) adj1 learning) or AI).tw,kf. | 311155 |
| 12 | 8 or 9 or 10 or 11 | 657657 |
| 13 | 7 and 12 | 26 |
| **Scopus** | | |
| 1 | TITLE-ABS-KEY ( "glaucoma surg*" OR trabeculectomy* OR migs ) AND TITLE-ABS-KEY ( outcome* ) AND TITLE-ABS-KEY ( "artificial intelligence" OR "machine learning" OR "deep learning" OR ai ) | 18 |
| **Web of Science** | | |
| 1 | (TS=("glaucoma surgery" OR "glaucoma surgical" OR "trabeculectomy" OR "migs") OR TI=("glaucoma surgery" OR "glaucoma surgical" OR "trabeculectomy" OR "migs")) | 19,901 |
| 2 | (TS=("outcome") OR TI=(“outcome”)) | 3,628,679 |
| 3 | (TS=("artificial intelligence" OR "machine learning" OR "deep learning" OR "AI") OR TI=("artificial intelligence" OR "machine learning" OR "deep learning" OR "AI")) | 2,006,901 |
| 4 | 1 AND 2 AND 3 | 25 |
