# Supplementary material for: Artificial intelligence for surgical outcome prediction in glaucoma: a systematic review
Source: Front Big Data. 2025 Aug 8;8:1605018. doi: 10.3389/fdata.2025.1605018 (PMC12370750; doi:10.3389/fdata.2025.1605018)
Supplement: Supplementary file 2 [file Table_2.docx]

**Supplementary Table 2.** Data Extraction Table

| **Study ID** | **Title** | **Objective** | **Study Design** | **Setting** | **Population Description** | **# of Patients** | **# of Surgeries** | **Age** | **Sex** | **Race** | **Inclusion Criteria** | **Exclusion Criteria** | **Data Source** | **Surgical Intervention** | **Surgical Failure Criteria** | **Surgical Success Criteria** | **Outcome(s)** | **Follow-Up Time** | **Modeling Approach** | **Best Performing Model** | **Features** | **Variable Importance Ranking** | **Data Imbalance** | **Internal Validation** | **External Validation** | **Accuracy** | **AUROC/AUC** | **AUPRC** | **ACC** | **MCC** | **F1 Score** | **Precision** | **NPV** | **Sensitivity (Recall)** | **Specificity** | **Conclusions** | **Limitations** |
| --- | --- | --- | --- | --- | --- | --- | --- | --- | --- | --- | --- | --- | --- | --- | --- | --- | --- | --- | --- | --- | --- | --- | --- | --- | --- | --- | --- | --- | --- | --- | --- | --- | --- | --- | --- | --- | --- |
| Agnifili 2023 |  |  |  |  |  |  |  |  |  |  |  |  |  |  |  |  |  |  |  |  |  |  |  |  |  |  |  |  |  |  |  |  |  |  |  |  |  |
| Banna 2023 |  |  |  |  |  |  |  |  |  |  |  |  |  |  |  |  |  |  |  |  |  |  |  |  |  |  |  |  |  |  |  |  |  |  |  |  |  |
| Barry & Wang 2024 |  |  |  |  |  |  |  |  |  |  |  |  |  |  |  |  |  |  |  |  |  |  |  |  |  |  |  |  |  |  |  |  |  |  |  |  |  |
| Birla 2024 |  |  |  |  |  |  |  |  |  |  |  |  |  |  |  |  |  |  |  |  |  |  |  |  |  |  |  |  |  |  |  |  |  |  |  |  |  |
| Lee 2024 |  |  |  |  |  |  |  |  |  |  |  |  |  |  |  |  |  |  |  |  |  |  |  |  |  |  |  |  |  |  |  |  |  |  |  |  |  |
| Lin 2024 |  |  |  |  |  |  |  |  |  |  |  |  |  |  |  |  |  |  |  |  |  |  |  |  |  |  |  |  |  |  |  |  |  |  |  |  |  |
