# Supplementary figures and images for: Artificial intelligence for surgical outcome prediction in glaucoma: a systematic review
Source: Front Big Data. 2025 Aug 8;8:1605018. doi: 10.3389/fdata.2025.1605018 (PMC12370750; doi:10.3389/fdata.2025.1605018)

**Supplementary Figure 1. PRISMA Flow Chart**

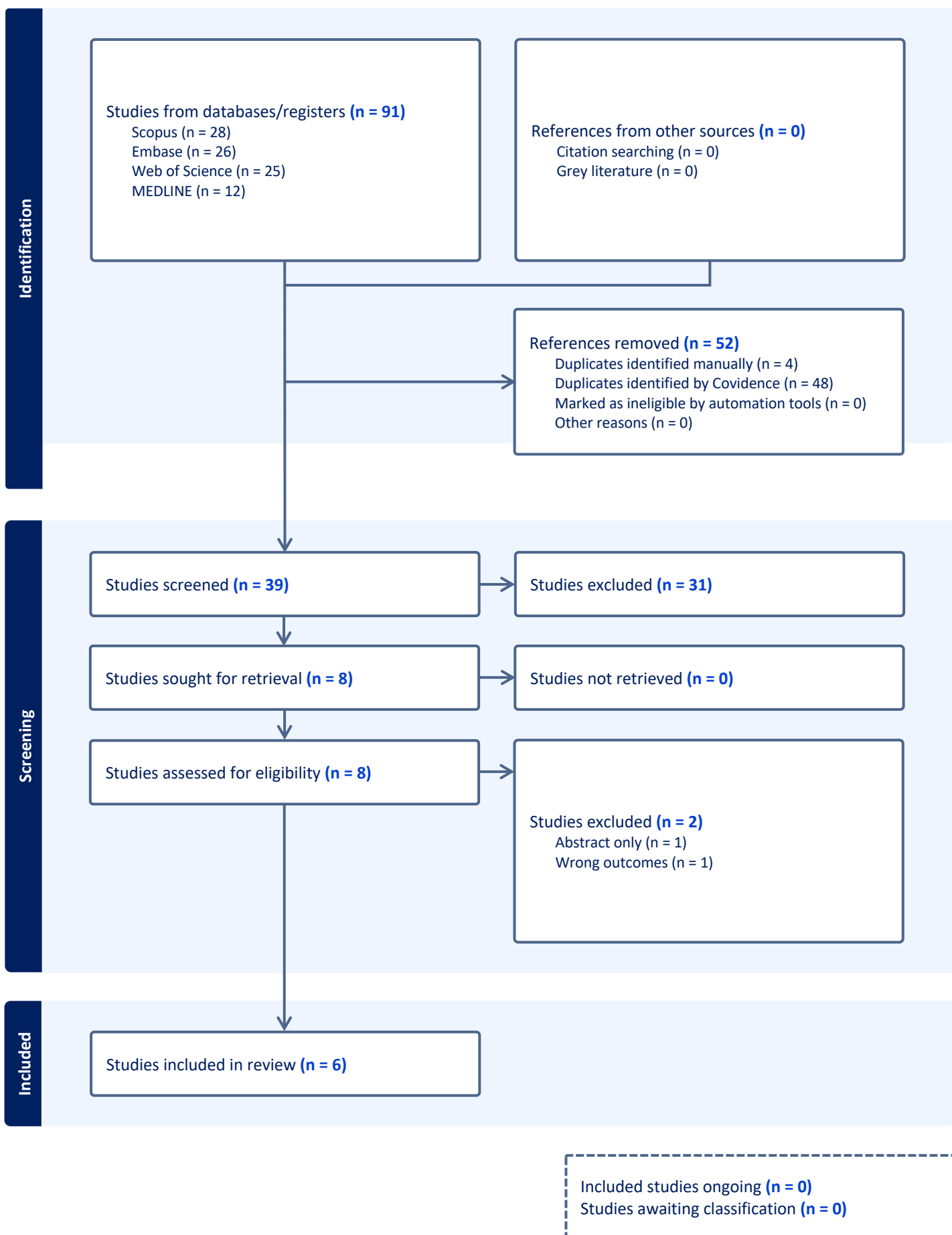

Supplement: Supplementary file 3 [file Image_1.pdf]
